# The evolution of leader–follower reciprocity: the theory of service-for-prestige

**DOI:** 10.3389/fnhum.2014.00363

**Published:** 2014-06-04

**Authors:** Michael E. Price, Mark Van Vugt

**Affiliations:** ^1^Department of Psychology, School of Social Sciences, Brunel UniversityUxbridge, UK; ^2^Faculty of Psychology and Education, VU University AmsterdamAmsterdam, Netherlands; ^3^University of OxfordOxford, UK

**Keywords:** leadership, followership, reciprocity, collective action, evolutionary psychology, social status, dominance, prestige

## Abstract

We describe the service-for-prestige theory of leadership, which proposes that voluntary leader–follower relations evolved in humans via a process of reciprocal exchange that generated adaptive benefits for both leaders and followers. We propose that although leader–follower relations first emerged in the human lineage to solve problems related to information sharing and social coordination, they ultimately evolved into exchange relationships whereby followers could compensate leaders for services which would otherwise have been prohibitively costly for leaders to provide. In this exchange, leaders incur costs to provide followers with public goods, and in return, followers incur costs to provide leaders with prestige (and associated fitness benefits). Because whole groups of followers tend to gain from leader-provided public goods, and because prestige is costly for followers to produce, the provisioning of prestige to leaders requires solutions to the “free rider” problem of disrespectful followers (who benefit from leader services without sharing the costs of producing prestige). Thus service-for-prestige makes the unique prediction that disrespectful followers of beneficial leaders will be targeted by other followers for punitive sentiment and/or social exclusion. Leader–follower relations should be more reciprocal and mutually beneficial when leaders and followers have more equal social bargaining power. However, as leaders gain more relative power, and their high status becomes less dependent on their willingness to pay the costs of benefitting followers, service-for-prestige predicts that leader–follower relations will become based more on leaders’ ability to dominate and exploit rather than benefit followers. We review evidential support for a set of predictions made by service-for-prestige, and discuss how service-for-prestige relates to social neuroscience research on leadership.

## INTRODUCTION

Leadership and followership have evolved to facilitate information sharing and coordinated group action in a wide variety of species (King et al., 2009). Humans are apparently adapted for complex cooperative behaviors that require high levels of expertise, coordination, and solutions to collective action problems (Tooby et al., 2006), and it would not be surprising if they, like so many other species, have also evolved psychological adaptations for leadership and followership (Van Vugt and Ahuja, 2010; Van Vugt and Ronay, 2014). In this article, we propose that such adaptations have indeed evolved, and that they govern the dynamics of leader–follower relations in human organizations. Our focus is specifically on leader–follower relations in humans, as opposed to any other species. We describe a theory of leader–follower relations which we think will ultimately enhance social neuroscientists’ understanding of the neural processes that enable these relations. Scientists are still in the early stages of understanding how the mind is adapted to lead and follow, and of developing neuroscientific methods for identifying psychological adaptations (Van Vugt, 2014). Nevertheless, the key conceptual elements of a coherent and plausible evolutionary theory of leader–follower relations are already in place (Price and Van Vugt, in press), and neuroscientists have already begun using evolutionary theories of psychological adaptation to guide their research on social interactions (Rilling and Sanfey, 2011). Thus, we propose that evolutionary social psychologists and social neuroscientists should begin engaging with each other more on the topic of leader–follower relations, and thinking about ways in which evolutionary approaches to these relations could both inform and be informed by neuroscientific research.

From the perspective of evolutionary psychology (Tooby and Cosmides, 1992, 2005), voluntary social relationships are likely to evolve if they provide all partners in the relationship with individual fitness benefits – that is, with benefits that enhance the survival and reproduction of the individual (and/or the individual’s very close genetic kin). We take this perspective on leader–follower relations, so a key question driving our analysis is: how might leader–follower relationships have been mutually fitness-enhancing for both leaders and followers in the environments of the evolutionary past? We pay close attention to past evolutionary environments, because any evolved psychological mechanisms that exist today in the minds of modern humans, including those governing leader–follower relationships, could exist only if they functioned adaptively in these environments (Tooby and Cosmides, 1990).

We propose that voluntary leader–follower relationships – that is, interactions in which followers voluntarily follow, and leaders voluntarily lead, because they each perceive some positive incentive to do so – were adaptive in the past for both leaders and followers because they involved mutually beneficial exchange. In this exchange, leaders enhanced the fitness of followers by providing them with collectively shared benefits and resources (often in the form of “public goods”) that enhanced followers’ wealth, status, and ability to function in coordinated and cooperative groups, and followers enhanced the fitness of leaders by providing them with prestige. As we will discuss in more detail below, by “prestige” we mean social status that is voluntarily conferred on those who are useful to others, as distinguished from “dominance,” which is status that is attained coercively by those who are threatening to others (Henrich and Gil-White, 2001). Evidence suggests that prestige and dominance are two distinguishably different paths that individuals can take in order to increase their social status (Von Rueden et al., 2011; Cheng et al., 2012).

The more equal the social bargaining power of leaders and followers (i.e., the more equal their abilities to confer benefits and/or impose costs on one another; Sell et al., 2009), the more likely the leader–follower relationship would have remained voluntary, mutually fitness-enhancing, and maximally beneficial overall. However, the greater the bargaining power of leaders relative to followers, the more likely the relationship would have been to transition from being reciprocal and prestige-based to being coercive, exploitative, and dominance-based. This transition should occur because from the leader’s perspective, the leader–follower relationship is advantageous primarily as a means of acquiring and maintaining high social status. When followers have relatively high relative bargaining power (e.g., high freedom to exit the group, high power to reject or retaliate against leaders), the easiest way for leaders to achieve high status will be to make themselves useful to followers by offering them benefits in exchange for prestige. In these situations, if leaders attempt to claim high status without offering followers anything in return, or by attempting to dominate and coerce followers, then their would-be followers can simply reject them (e.g., exit the group or depose the leader). However, when followers have relatively low bargaining power, leaders will have increased ability to gain and maintain status based on their ability to dominate, rather than benefit, followers. For example, if followers have low power to exit a group or to strip a leader of his or her high status, then the leader will have little need to offer them benefits, in order to compel them to (a) stay in the group or (b) grant the leader high status in exchange for these benefits. Leaders may sometimes perceive dominance, as compared to reciprocity, to be an appealingly cheap and efficient route to high status, as it saves them the costs of having to produce benefits for followers.

We refer to the above theory of how and why leader–follower relationships vary from reciprocity to dominance as *service-for-prestige* (Price and Van Vugt, in press).

### HOW ARE LEADER–FOLLOWER RELATIONS DIFFERENT IN HUMANS THAN IN OTHER SPECIES?

As noted above, leader–follower relations have evolved in a wide variety of species to allow individuals to share information and coordinate their behavior (King et al., 2009). For instance in many taxa, individuals share knowledge in order to lead followers to the locations of food, water, and other resources (examples include ravens, elephants, and most famously honeybees, who map out directions to resources via waggle dances); in many fish species, leader–follower dynamics result in groups (shoals and schools) that are helpful for avoiding predators and finding food; and among some primates such as chimpanzees, alpha males lead aggressive group actions against enemy groups and predators (Boehm, 1999; Krause and Ruxton, 2002; King et al., 2009). In the human lineage, just as in other species, leadership probably evolved initially to solve problems related to information sharing and social coordination. However, we propose that eventually, evolution enabled humans to use reciprocity to enhance the benefits of leadership.

To understand why reciprocity could enhance leadership, consider that leader–follower dynamics often evolve in situations where individuals are better off acting in groups as opposed to acting alone, for example, because group membership increases one’s likelihood of finding resources or escaping predators (Hamilton, 1971; Van Vugt and Kurzban, 2007). Such group movements present coordination problems, however, associated with determining who will lead and who will follow. For example, if Individuals A and B both have an interest in visiting a waterhole together (because there is safety in numbers), and have several waterholes to choose from, how will they choose which one to visit? There are several ways in which leader–follower dynamics could emerge to solve this problem (Van Vugt and Kurzban, 2007). For example, imagine that A prefers a particular waterhole but B has no preference, and as a result A moves first to choose the preferred waterhole. Once A has made this move, B is best off following A, as opposed to making a dangerous solo journey to a waterhole that offers B no additional benefits. Leadership may have evolved in many species to solve coordination problems such as these, when there has been a fitness advantage to the individual in assuming a leadership role (Van Vugt, 2006).

However, what about situations in which the individual is disadvantaged by assuming a leadership role? Many leadership roles may involve substantial costs to leaders, and individuals may need special incentives to accept these roles. If followers stand to benefit from a leader’s assumption of a costly role (e.g., if this leadership would provide protection for followers), then it might profit followers to provide the leader with these incentives. This potential for reciprocity could provide new opportunities for leadership to evolve, such that leader–follower relations could become not just matters of coordination but also matters of exchange. We propose that leader–follower relations evolved as service-for-prestige transactions in contexts such as these, to enable leadership behaviors that would otherwise have been prohibitively costly. However, engagement in reciprocity, particularly in the complexly cooperative social environments of human beings, requires specially designed social-cognitive abilities that are uniquely sophisticated in humans (Tooby and Cosmides, 1996; Hammerstein, 2002; Tooby et al., 2006; Bowles and Gintis, 2011). Therefore, we propose that service-for-prestige exchange is a crucial aspect of leader–follower dynamics in humans, but not necessarily in any other species.

## THEORETICAL FOUNDATIONS OF SERVICE-FOR-PRESTIGE: SYNTHESIZING THEORIES OF RECIPROCITY AND COLLECTIVE ACTION, AND ACCOUNTING FOR EVOLUTIONARY MISMATCH

### EVOLUTIONARY THEORIES OF RECIPROCITY

Human leader–follower relationships are cooperative interactions that occur between people who are not necessarily close genetic kin. One of our key theoretical tools, therefore, will be the main concept used by evolutionists to explain non-kin cooperation: reciprocity (Trivers, 1971; Alexander, 1979; Tooby et al., 2006). Reciprocity theories assume that because cooperative individuals incur fitness costs in order to deliver fitness benefits to others, they must receive some return benefit from others as compensation for these costs. In the absence of such compensation, cooperation will be maladaptive for cooperators and will not evolve.

The most basic form of reciprocal cooperation is direct reciprocity, described originally by Trivers (1971) as “reciprocal altruism.” Trivers (1971) described mutually beneficial exchange between a cooperator (or “altruist”) and a reciprocating partner. For example, if X pays a cost of size 1 to provide Y with a benefit of size 2, and Y precisely returns the favor, then X and Y will each have paid a cost of 1 and received a benefit 2, and the exchange will be mutually profitable. However, it is crucial to note that Y could have profited even more by “cheating,” that is, by taking X’s 2 without reciprocating 1. In order for reciprocity to evolve in direct exchange contexts, cooperators must somehow avoid being exploited by cheaters, for example, by avoiding them altogether, or by neutralizing their advantage via punishment. If cheaters consistently tend to come out ahead in these interactions, they will eventually exploit cooperators to extinction and cooperation will not evolve (Hamilton, 1964; Trivers, 1971; Henrich, 2004; Price and Johnson, 2011). Individuals should thus be predisposed to cooperate with reciprocators, and be averse to cooperating with cheaters. This prediction is supported by a large body of evidence from several behavioral science fields (Price, 2006a). Reciprocity has long been considered a fundamental attribute of human social systems cross-culturally (Gouldner, 1960), and it is generally considered to be a universal, species-typical, and highly fitness-relevant human behavior (Brown, 1991; Rilling and Sanfey, 2011).

The reciprocity theory presented by Trivers (1971) primarily describes reciprocity that is direct and dyadic (i.e., involving direct exchange between two individuals). However, extensions of this theory have been used to explain other forms of reciprocity. The best known example is “indirect reciprocity,” that is, interactions in which X’s altruism toward Y is reciprocated not by Y but by a third party (Alexander, 1979; Nowak and Sigmund, 2005). There have also been attempts to apply reciprocity theory to direct exchanges between one individual and a group of other individuals (Boyd and Richerson, 1988; Price, 2003, 2006a; Tooby et al., 2006; Takezawa and Price, 2010). Because leader–follower relations often (although not exclusively) involve interaction between one leader and multiple followers, this kind of reciprocity would seem most relevant to an understanding of leader–follower exchange. However, it is not widely accepted among evolutionary researchers that direct reciprocity can explain the evolution of cooperation in group contexts such as these (Boyd and Richerson, 1988; Henrich, 2004; Bowles and Gintis, 2011). In the view of these researchers, direct reciprocity can explain the evolution of simple dyadic cooperation, but totally different processes such as cultural group selection are required to explain cooperation in groups. Our application of reciprocity to these contexts, then, does not represent the consensus view of evolutionary researchers, and important theoretical questions still need to be resolved about precisely how leader–follower exchange could evolve.

Nevertheless, despite this lack of a theoretical consensus, we agree with previous suggestions (Price, 2003, 2006a; Tooby et al., 2006) that direct reciprocity (in combination with indirect reciprocity) may be a key factor in the evolution of group cooperation in humans, and we do not think that it is premature or implausible to suggest that leaders and followers often engage in mutually beneficial exchange. We propose that reciprocity theory provides the most appropriate and predictive evolutionary framework for understanding voluntary human leader–follower interactions, because (1) leaders often incur costs in their efforts to provide benefits for followers, (2) followers often incur costs in order to provide prestige which benefits leaders, (3) in order for each of these costly provisioning behaviors to be adaptive in the ancestral past, both leaders and followers would have needed to recoup these costs somehow, and (4) this recoupment could plausibly have occurred via a process in which leader-produced benefits were exchanged for follower-produced prestige. Illustrations of why it is often costly for leaders to provide public goods and for followers to provide prestige, and of why prestige entails fitness benefits, are presented below.

### LEADER–FOLLOWER RECIPROCITY AS A COLLECTIVE ACTION PROBLEM

Our second key theoretical tool is Olson’s (1965) theory of collective action, which states that even if group members benefit on average if their group cooperates effectively, individual members can often reap the greatest private profits if they “free ride” while the rest of the group pays the costs of cooperation. This free rider problem – the private incentive that each individual member has to free ride on everyone else’s contributions – often seriously undermines group efforts to cooperate, and is considered by behavioral scientists to be the fundamental obstacle to successful collective action (Yamagishi, 1986; Boyd and Richerson, 1988, 1992; Ostrom, 1990).

Service-for-prestige regards leader–follower reciprocity as a collective action problem because many benefits provided by a leader (e.g., increased group status, improved group defense, and access to resources) will be public goods, shared widely and more or less equally by followers. A leader’s motivation to provide such benefits will thus also constitute a (second-order) public good. The public goods provided by the leader will often be costly to produce, and if increased prestige is what motivates the leader to pay these production costs (as service-for-prestige predicts), then followers must succeed in providing the leader with prestige, in order to maintain production of these public goods. If the prestige allocated to leaders is costly for individual followers to provide, then its allocation will present a collective action problem for followers (Price, 2003; Price and Van Vugt, in press).

To understand why prestige allocation should constitute a collective action problem for followers, it helps to first consider social power more abstractly. Emerson (1962) provides a simple and useful definition of social power when he notes the reciprocal relationship between power and dependence: Individual X has power over Individual Y to the extent that Y must depend exclusively on X for the achievement of some goal, that is, to the extent that Y’s ability to achieve the goal is controlled by X. Similarly, because Y’s goals will generally involve the acquisition of benefits and avoidance of harm, the power of X (i.e., the dependence of Y on X, and X’s control over Y’s goal achievement) can also be thought of as X’s ability to confer benefits and/or impose harm on Y (Sell et al., 2009). If X has high power, this should improve X’s access to many kinds of resources: if X is highly able to benefit and/or harm others, then those whom X can benefit/harm will be motivated to act in ways that promote X’s welfare, so that they can remain in good standing with X and thus acquire these benefits and/or avoid this harm. As a result, people will tend to go out of their way to promote X’s welfare (Henrich and Gil-White, 2001; Sell et al., 2009), for example, by deferring to X’s interests, sharing resources with X, taking pains to avoid causing harm to X, and cooperating with X. High social power (“status”) is therefore expected to benefit X’s fitness by improving X’s access to many kinds of resources (Von Rueden et al., 2011, in press; for more on the nature of these resources, see below section on small-scale societies).

The notion that social power is rooted in ones’ ability to benefit and/or harm others corresponds well to Henrich and Gil-White’s (2001) conceptualization of prestige and dominance as two different kinds of social status (noted above), with prestige being status that is voluntarily conferred on those who are perceived as offering benefits, and dominance being status that is attained coercively by those who are perceived as threatening harm. (However, note that these two paths to status will rarely be completely distinct, since traits that lead to prestige can frequently lead to dominance, and vice versa; Henrich and Gil-White, 2001; Von Rueden et al., 2011). If prestige is conceptualized as something that is freely conferred, then efforts made by the prestige allocator to promote the prestigious individual’s welfare ought to be thought of – like any kind of behavior in which one individual intentionally and voluntarily incurs costs to deliver benefits to another individual – as a kind of biological “altruism” (Tooby and Cosmides, 1996). We believe that conceptualizing prestige in this way, and distinguishing it from dominance, are useful means of understanding the different ways in which leaders can acquire status. However, unlike the service-for-prestige theory we present here, Henrich and Gil-White (2001) regard prestige as something that is offered in exchange not for public goods but for a private good: the privilege of affiliating socially with the prestigious individual. In their view, individuals with high levels of expertise are allocated prestige by those who wish to learn from and copy their behavior; by allocating prestige to an expert (i.e., by acting in ways that benefit the expert’s welfare), followers can ingratiate themselves to, and thus enhance their own ability to copy, the expert. Although we do agree that prestige allocation may often occur as a way to compensate experts for providing private goods, we suggest that it also often occurs as a way to compensate leaders for providing public goods.

If prestige is indeed allocated in exchange for public goods, and if prestige and its behavioral consequences are indeed costly to produce, then it becomes easy to see why the allocation of prestige to leaders will entail a collective action problem. In order for followers to motivate leaders to provide public goods, they must collectively pay the costs of respect. That is, they must as a group incur costs to allocate prestige to the leader – and the increased access to the group’s material, reproductive, and social resources that this prestige will entail – to an extent that compensates the leader for the cost of providing public goods. Because this represents a collective action problem, a follower could gain a free rider’s advantage by accepting the benefits of leadership while refusing to pay these costs. For example, consider a leader who routinely incurs costs (e.g., risks his own life in battle, assumes stressful responsibilities, works long and hard on military strategy) in order to guide his group to success in war. His services enable his followers to acquire public goods such as better territory, shared resources, and increased group status. Imagine two followers in this group, both of whom benefit equally from the leader’s services. Follower 1 is respectful, and tends to engage in costly acts that benefit the leader (e.g., does favors for and shares resources with the leader; refrains from having an affair with the leader’s wife; takes pains to look out for the welfare of the leader’s children; strives to comply with the leader’s directives; pays taxes or tribute to the leader; takes risks to ensure the safety and health of the leader). Follower 2 free rides on the leader’s services by doing none of these things, and thus enjoys higher net benefits (benefits received from the leader’s services, minus costs paid to be respectful) than Follower 1. Because each follower in this scenario has a personal incentive to free ride, there is the risk that the collective effort will fail to produce sufficient prestige to compensate the leader for the costs of providing public goods.

Just like cheaters in reciprocal exchanges, free riders in collective actions will exploit cooperators to extinction unless their advantages are neutralized (Yamagishi, 1986; Boyd and Richerson, 1992). As a result, cooperators strive to neutralize free riders’ advantages via punishment or social exclusion (Fehr and Gächter, 2002; Price and Johnson, 2011). The collective action scenario described here is unusual in that it is a collective action for the purpose of engaging in reciprocity. Collective actions are typically conceptualized as functioning to produce or acquire some shared material resource (for example, a group of citizens jointly generating tax revenue, or a group of hunters jointly killing a large game animal), but in this case, the joint effort is focused on producing sufficient prestige to compensate the leader for services rendered. As a result, Follower 2 above is in the unusual position of simultaneously representing both a cheater in a reciprocal interaction (for failing to engage in a service-for-prestige transaction with the leader) and a free rider in a collective action (for failing to cooperate with fellow followers in collectively producing prestige for the leader). Follower 2 will therefore be a prime target for hostility within the group: both the leader and the other followers have incentives to punish or ostracize Follower 2.

Because service-for-prestige is unique (as far as we know) in regarding the allocation of prestige to leaders as a collective action problem, it is also unique in predicting that this problem will need to be solved via the punishment and/or social exclusion of disrespectful followers. We say more about this prediction and related predictions below.

### MISMATCH THEORY: ANCIENT ADAPTATIONS IN MODERN ENVIRONMENTS

A final key component of service-for-prestige is mismatch theory. Because psychological adaptations evolved in ancestral environments that may be quite different in certain respects than present environments, we cannot always expect for adaptations to function adaptively in modern societies (Tooby and Cosmides, 1990). A common example is human gustatory preferences for fats, sugars, and salts. Because these nutrients were essential but difficult to acquire in ancestral environments, people have apparently evolved to be strongly motivated to consume them. These motivations may function maladaptively in environments where these nutrients are easily obtained, by leading to health problems associated with over-consumption (Nesse and Williams, 1994). Some aspects of leader–follower relations may represent mismatches with modern environments (Van Vugt et al., 2008b); we provide several examples below.

## LEADER–FOLLOWER RELATIONS IN THE HUMAN EVOLUTIONARY PAST

Before we focus on service-for-prestige in modern contexts, we will examine how leadership and followership operated in small-scale (i.e., hunter-gatherer and tribal) societies that most closely approximate those in which our ancestors spent the vast fraction of their evolutionary history.

The available evidence suggests that leadership and followership are universal aspects of human nature: these behaviors appear at all levels of social organization that have existed since prehistoric times, including hunter-gatherer and tribal societies (Brown, 1991; Van Vugt et al., 2008a). Leadership is used in these societies to facilitate cooperation in activities such as warfare, forging political alliances, maintaining within-group order, big game hunting, and moving camp (Service, 1966; Johnson and Earle, 1987), all of which are vital to the success, status, and fitness of individuals living in groups. Ethnographic accounts of leaders in these domains generally describe the leaders as men, rather than women (Service, 1966; Johnson and Earle, 1987). However, although women only rarely hold the most directly influential political positions in small-scale societies, they commonly lead in more indirect ways by exerting substantial influence on political affairs (Low, 1992; Yanca and Low, 2004; Bowser and Patton, 2010).

A common observation about leadership in small-scale societies is that it tends to be informal and based on achievement (Fried, 1967; Kelly, 1995). Compared to leaders in industrialized societies, these leaders have little power to force others to do what they say. This is especially true in nomadic hunter-gatherer societies, which, compared to sedentary small-scale societies, involve smaller group sizes and lower population densities. There are usually no formal leadership offices or duties in nomadic hunter-gatherer societies, and leaders tend to lead by persuasion and demonstrations of their expertise and ability to benefit others (Service, 1966; Johnson and Earle, 1987). Nomadic hunter-gatherers rarely recognize anyone as a formal headman and tend to express low tolerance for domineering leaders (Service, 1966; Turnbull, 1968; Lee, 1993).

Small-scale societies also tend to recognize different leaders in different domains (cf. the concept of distributed leadership; Gronn, 2002). Leadership requires expertise, and different people may have expertise in different activities (Service, 1966). For instance, the leader of a hunting expedition may not be the same person who organizes an alliance with a friendly group or a raid against an unfriendly one. The traditional authority system of the Navajo, for example, included war leaders, peace leaders (who organized friendly political interactions), hunt leaders, medical leaders, and ceremonial song leaders (Shepardson, 1963).

By assisting the group in domains such as political relations with external groups, maintenance of internal order, big game hunting, and camp movements, leaders provided followers with public goods. For instance, success in war can bring a wide variety of collective benefits, including increased access to territory, mates, and other resources (Keeley, 1996), and success in hunting large game produces meat that is widely shared among the entire residential group (Kelly, 1995). Leaders often incur large costs to generate these public goods. Big game hunting, for example, can involve large investments of time and effort and significant risks. A survey of “persistence hunts” among Kalahari hunter-gatherers suggests that members of hunting parties chase large game for 3–6 h across distances of 20–35 km (12–22 mi), in difficult conditions such as extreme heat and dense bush (Liebenberg, 2006). War leadership represents another example of costly public goods provisioning; war leaders gain reputations for bravery by taking risks (for example, fighting in the front lines) that enable their groups to effectively compete for resources (Meggitt, 1977; Chagnon, 1988).

Why are leaders willing to incur large costs in order to provide followers with public goods? Plausibly because provisioning of public goods is a key way in which members of small-scale groups can acquire social status (Price, 2003, 2006a, b). Because leaders in small-scale societies have little power to coerce and dominate followers, their high status appears to be more similar to voluntarily conferred prestige than to dominance (Henrich and Gil-White, 2001; Van Vugt and Ahuja, 2010). These leaders benefit from their high status: prestigious individuals are highly valued by others as friends and allies and therefore social and material resources tend to flow their way. Leaders’ increased access to these resources may sometimes be observable only over the long-term, as opposed to the immediate short-term (Von Rueden et al., in press); for example, among Ache forager-horticulturalists, those who consistently produce and share large amounts of food appear to be rewarded over the long-term by receiving more food from others when they are sick or injured (Gurven et al., 2000). In a community of Tsimane hunter-horticulturalists, the most prestigious and influential men did not receive more shared food over the short-term, but were more likely to receive social support (e.g., help with labor), food, and cash during times of crop failure (Von Rueden, 2011). Similarly, magnanimous leadership among the Martu Aborigines in Australia is believed to be rewarded over the long-term with social and political support (Bird and Bliege Bird, 2010).

A further important way in which status enhances male fitness in these societies is by contributing to reproductive success. Status is attractive both to women (Ellis, 1992; Li, 2007) and to parents who wish to betroth their daughter to a high status man as a way of creating a useful ally (Hart and Pilling, 1960; Kelly, 1995). Ethnographic evidence suggests that in these societies, higher status men – or leaders – have more wives and sexual partners, as well as higher-fertility wives and more surviving offspring (Levi-Strauss, 1967; Chagnon, 1979, 1988; Betzig, 1986; Von Rueden et al., 2008, 2011). For example, relatively high levels of status and reproductive success are attained both by leaders in the hunting domain who provide their group with large game as a public good (Hawkes, 1993; Hawkes and Bliege Bird, 2002), and by leaders in the war domain who contribute to their group’s ability to compete for resources (Matthiessen, 1962; Meggitt, 1977; Chagnon, 1988).

The importance of leadership in small-scale societies tends to correlate positively with the degree to which settlement patterns are sedentary as opposed to nomadic, because sedentism permits larger residential group sizes and higher population density (Fried, 1967; Johnson and Earle, 1987; Marlowe, 2011). When groups are larger, coordination and collective action problems involved in group action are harder to solve, and leadership is relatively more important (Carneiro, 2000; Tooby et al., 2006; Hooper et al., 2010). Within-group disputes between members (e.g., dyadic conflicts) may also become more frequent in larger groups, necessitating leadership to resolve them. Further, when population density is lower and it’s easier to move camp, individuals can more freely leave and switch groups. Nomadic hunter-gatherers exhibit “fission–fusion” social organization, with unstable group membership rosters. Groups may break apart or join together, depending on the abundance of local resources and the quality of social relationships within the group (Turnbull, 1968; Kelly, 1995). This arrangement makes it relatively easy for group members to escape a leader who becomes too dominant. But with increases in sedentism and population density, fission–fusion social organization becomes less tenable, and followers become less capable of exiting groups with dominant leaders (Boehm, 1999; Price and Van Vugt, in press). Sedentism may also be associated with more powerful leadership if it is enabled by the presence of a “patchy” resource (i.e., one concentrated in a fixed location) that can be monopolized and controlled by leaders, such as the salmon runs of the Northwest Coast described below (Kelly, 1995).

The transition to agriculture is associated with increases in group size, population density, and sedentism, as well as increases in the power and dominance of leaders. Typical nomadic hunter-gatherer bands consist of 25–50 members, but typical hunter-horticulturalist villages consist of 100–400 residents (Johnson and Earle, 1987; Kelly, 1995). Leaders are more powerful in hunter-horticultural compared to nomadic hunter-gatherer societies, with formally recognized “Big Men” exhibiting enduring political authority, and with social organization becoming more hierarchical (Meggitt, 1977; Johnson and Earle, 1987; Chagnon, 1997; Boehm, 1999). Greater sedentism and population density also mean that hunter-horticultural settlements are more “socially circumscribed” (i.e., hemmed in by neighboring settlements) than the camps of nomadic hunter-gatherers (Chagnon, 1997), which reduces the feasibility of fission–fusion organization and the ability of followers to escape overly dominant leaders.

However, it is not agriculture *per se*, but rather the increased group size and population density that agriculture permits, that seems to lead to increases in the power and dominance of leaders. The Indians of the American Pacific Northwest Coast provide a useful illustration of how leaders can become more powerful and dominant with increases in group size and population density, even in the absence of agriculture (Price and Van Vugt, in press). By residing near salmon-rich rivers, these hunter-gatherers could maintain sedentary villages of 500–800 people and population densities of one to two people per square mile, both unusually high figures for either hunter-gatherers or hunter-horticulturalists (Johnson and Earle, 1987). These villages required strong leaders because it is challenging to organize large groups for collective action and resource redistribution (Fried, 1967; Johnson and Earle, 1987). Accordingly, Northwest Coast leaders were much more powerful than typical hunter-gatherer leaders, and are regarded by anthropologists as being the key to the functioning of the Northwest Coast political economy (Johnson and Earle, 1987). These leaders were clearly identified by followers as chiefs and as essential group representatives in political interactions, and they broadcast their wealth and status in lavish potlatch ceremonies in which they distributed and sometimes destroyed large collections of their material goods.

Not only were followers more dependent on leaders in the environments of the Northwest Coast, they were also more helpless to escape dominant leaders, due to the unfeasibility of fission–fusion organization (that is, the sedentary lifestyle and high population density made it more difficult for group members to hive off from groups with bad leaders, in order to live and forage in a different territory). Moreover, the patchy distribution of salmon runs enabled chiefs to control access to the region’s most important food resource, which further increased follower dependence (Kelly, 1995). The decreased exit options and increased dependence of followers seem to have increased the extent of dominance-based leader–follower relationships: although slavery is rare in hunter-gatherer societies, it was common throughout the Northwest Coast, with slaves composing 7–15% of a typical community (Kelly, 1995). As noted above, this association between reduced follower bargaining power and increased dominance in leader–follower relations is a prediction of service-for-prestige, which assumes that leaders tend to maintain their high status in the least costly way that they can; if they can maintain it without having to pay the costs of providing benefits for followers, they will tend to do so. That is, when followers are more powerless to escape, reject, or retaliate against leaders, leaders will more likely attempt to maintain their status via their ability to dominate and exploit followers, as opposed to their ability to engage in reciprocity with them.

The positive relationships between group size/population density and more powerful leadership can also be observed not just by comparing different societies but by comparing different settlement patterns within the same society. Carneiro (2000) describes how these patterns changed seasonally among North American Plains Indians. For most of the year they lived in small bands of about 50 people, but during the summer buffalo hunt 20 or more of these bands would coalesce to form a much larger group. The dramatic size increase was accompanied by an equally dramatic elaboration of leadership structure. Whereas leadership in the single band involved little power and few duties (i.e., it was fairly typical of a small nomadic foraging society), leadership in the large aggregation involved a tribal council of band leaders headed by a designated tribal chief, as well as several men’s societies, including one that acted as a police force to maintain order in the settlement.

Like the different small-scale societies discussed above, the hunter-gatherer societies of the human evolutionary past have probably varied considerably in terms of key demographic factors such as group size and population density (Kelly, 1995). We suggest that this variation affected the balance of power between leaders and followers and therefore influenced the prevalence of reciprocal versus coercive leadership of these societies. We also suggest that human mental adaptations for leadership and followership were designed by the selection pressures that existed across this range of different environments, and so are calibrated to generate a range of behavioral outputs, depending on the balance of power between leaders and followers that is perceived in the environment.

## SERVICE-FOR-PRESTIGE IN INDUSTRIALIZED SOCIETIES

The human mind was designed by and for the environments of small-scale ancestral societies (Tooby and Cosmides, 1990, 1992, 2005). The above examination of leader–follower relations in such societies therefore provides an essential foundation for our next task, which is to evaluate the extent to which predictions of service-for-prestige are supported by observations of leader–follower relations in industrialized societies.

### PREDICTION 1: FOLLOWERS PREFER TO CHOOSE THEIR OWN LEADERS, BY ALLOCATING PRESTIGE TO THOSE WHO PROVIDE THEM WITH BENEFITS

Experimental evidence suggests that people in industrialized societies, just like in small-scale societies, prefer to follow leaders who they have chosen themselves, rather than leaders who have been imposed on them by an external agent (Van Vugt et al., 2004). Further, their mechanism for choosing leaders is similar to that used in small-scale societies (Price, 2003, 2006a, b): they reward group-beneficial contributions with prestige (Price and Van Vugt, in press). Studies conducted among both university students and business employees indicate that when group members are allowed to allocate status to the co-members of their choosing, they allocate it to those who have demonstrated their willingness and ability to benefit the group (Flynn, 2003; Hardy and Van Vugt, 2006; Anderson and Kilduff, 2009; Willer, 2009). This process of status acquisition via engagement in group-beneficial tasks has been termed “competitive altruism” (Roberts, 1998; Barclay, 2004; Hardy and Van Vugt, 2006; McAndrew and Perilloux, 2012): because group members obtain an individual advantage by achieving high status, and because status can be acquired via pro-group altruism, members compete with each other to be the most group-beneficial.

Representative governments (e.g., forms of democracy) are also characterized by processes whereby followers can choose their own leaders, and award prestige to leaders who benefit them (e.g., by voting them into office). Political philosophers have long noted that these processes are key reasons why representative governments tend to be more effective in delivering benefits to citizens, compared to less reciprocal arrangements such as monarchy (Locke, 1689; Mill, 1861). In most businesses, however, such democratic processes are absent, and leaders are simply imposed on followers. Thus a central dynamic of leader–follower reciprocity – the process whereby followers choose their leaders, and allocate prestige to them based on their ability to provide group benefits – cannot occur in most businesses, which probably results in followers becoming alienated and losing motivation to cooperate voluntarily with leaders (Price and Van Vugt, in press). Some successful businesses, however, are exceptions to this rule. Leaders at W. L. Gore and associates, for example, are selected via a process in which employees choose who they want to follow, rather than one in which bosses are imposed on employees. The philosophy behind this practice – “if you attract followers, then you’re a leader” – recalls the bottom-up process of leader selection that prevails among nomadic hunter-gatherers. W. L. Gore’s very low employee turnover rate suggests that they are implementing this practice to good effect (Van Vugt and Ahuja, 2010).

### PREDICTION 2: PREFERENCES FOR LEADERS WILL BE BIASED IN FAVOR OF PHYSICALLY FORMIDABLE AND INTELLIGENT MALES, AND MAY BE MISMATCHED WITH MODERN ENVIRONMENTS

Because of processes in sexual selection (Darwin, 1871; Trivers, 1972), men are on average more physically formidable (e.g., taller and stronger) than females. In the ancestral-type environments of small-scale societies, some of the most important domains in which leadership is required are male-dominated activities requiring high physical formidability, such as hunting and war. The leaders who are chosen in such domains are almost always males (Service, 1966; Johnson and Earle, 1987). As a result, our minds may have evolved to be biased toward assuming that all else equal, physically formidable males make the most appropriate leaders (Van Vugt and Ahuja, 2010). (Indeed more generally, people seem to cognitively encode the concept of political power as a human body, with higher power represented by a more formidable body; Holbrook and Fessler, 2013).

Followers do appear to exhibit such biases in industrialized societies: experimental and field studies of general leadership preferences suggest that people tend to prefer leaders who are male (Carlson et al., 2006; Elsesser and Lever, 2011), who are perceived as taller based on their stature or facial height (Judge and Cable, 2004; Gawley et al., 2009; Blaker et al., 2013; Re et al., 2013), and who are perceived as healthier based on their bodily motion (Kramer et al., 2010). Facial appearance may also provide cues to pubertal testosterone levels and thus to physical formidability. For example, male military academy graduates who were rated as appearing more dominant in their student portraits went on to achieve higher status in their careers (Mazur and Mueller, 1996). Another formidability-related cue is physical attractiveness; traits that are perceived as attractive are believed to be those which would have indicated health and biological quality in the ancestral past (Grammer et al., 2003). Accordingly, followers express preferences for physically attractive leaders (Anderson et al., 2001; Van Vugt and Ahuja, 2010).

Preferences for physically formidable male leaders may, however, be mismatched with leadership requirements in modern organizations (Van Vugt et al., 2008b). Our ancestors’ need for expertise in male-dominated, physically aggressive coalitional activities such as hunting and war is much-reduced in modern businesses, but biases in favor of physically formidable males persist. As a result of such biases, people in industrialized societies may, for reasons that have become largely obsolete, tend to overlook females and physically unimpressive males as candidates for leadership positions, even if these candidates are in fact well-suited for leadership in modern contexts (Van Vugt et al., 2008b; Price and Van Vugt, in press). On the other hand, even if there is less of a genuine need for physically formidable leaders in modern organizations than there was in the ancestral past, such leaders may nevertheless perform especially effectively in some kinds of leadership roles, due to how they are perceived by others. For example, a leader’s high formidability could increase his bargaining power in social interactions by making him seem more intimidating to others (Sell et al., 2009; Lukaszewski, 2013); as a result that leader might be relatively effective in tasks like deterring uncooperative behaviors among followers and winning negotiations with external groups.

Other leader traits that tend to be widely preferred are intelligence and communication skills (Den Hartog et al., 1999; Judge et al., 2004). These preferences also seem consistent with the requirements of leadership roles in the ancestral past (Tooby et al., 2006; Van Vugt et al., 2008a). For instance, intelligence is necessary for making decisions that will affect group welfare in a positive way (e.g., developing a strategy for successful group cooperative action), and communication skills are essential for implementing these decisions (e.g., persuading followers about the wisdom of that strategy, and ensuring that the group acts out that strategy in a precise and coordinated way). Unlike traits related to physical formidability, however, intelligence and communication skills are probably just as genuinely relevant to leadership roles in modern environments as they were in the ancestral past. Competent leadership in an industrialized society generally has little to do with killing large game or physically dominating rivals, but continues to have much to do with devising and communicating a successful strategy for collective action (Price and Van Vugt, in press).

### PREDICTION 3: PREFERENCES FOR LEADERS WILL BE DIFFERENT IN DIFFERENT DOMAINS, AND MAY BE MISMATCHED WITH MODERN ENVIRONMENTS

Leadership ability can be domain specific, and just as small-scale societies distinguish among several kinds of leaders (as noted above), members of industrialized societies prefer different leaders for different roles (Price and Van Vugt, in press). This may be a major reason why leadership is often shared in successful organizations (Wassenaar and Pearce, 2011). For example, experimental participants prefer leaders with a more masculine facial appearance (like John McCain) in the context of war and intergroup competition, and prefer leaders with a more feminine face (like Barack Obama) in peaceful contexts (Little et al., 2006; Spisak et al., 2012a, b). Domain-specific leadership preferences may even be strong enough to override the general bias in favor of male leaders, noted above: experimental evidence suggests that although male leaders are preferred in times of intergroup competition, female leaders are preferred when there is a need for conflict resolution within the group (Van Vugt and Spisak, 2008).

On the other hand, our bias toward domain-specific leadership may sometimes lead us astray, and can be another example of mismatch (Van Vugt and Ronay, 2014; Price and Van Vugt, in press). Hunter-gatherer collective actions tend to be small and therefore relatively simple to organize and manage, so domain-specific expertise (as opposed to management skills) may often be the most important requirement for competent leadership. It might make sense, for example, to choose the leader of a three-member hunting expedition based on hunting expertise. In a complex modern organization, however, the gulf between domain-specific expertise and leadership can be more vast, and managerial roles can require skills that have very little to do with this expertise itself. In many professional sports, for example, great former players are often preferred as managers, despite an absence of evidence that better players make better managers (Van Vugt and Ahuja, 2010). We should question our bias toward assuming that superior ability in a specific activity will make one particularly well-suited to lead an organization related to that activity.

### PREDICTION 4: FOLLOWERS WILL PREFER COMPETENT, PROSOCIAL, AND “FAIR” LEADERS, BUT SOME PREFERENCES MAY BE MISMATCHED WITH MODERN ENVIRONMENTS

According to service-for-prestige, followers are adapted to favor leaders who are willing and able to provide them with benefits (Price and Van Vugt, in press). Accordingly, research suggests that people from a wide variety of cultures do prefer leaders who score highly on both prosociality and competence (Van Vugt et al., 2008a). The 61-culture GLOBE survey of universally valued leader traits (Den Hartog et al., 1999) indicates that prosocial disposition (e.g., trustworthiness, fairness) and possession of group-beneficial skills (e.g., intelligence, competence) are consistently valued attributes of leaders cross-culturally. These findings complement a review by Hogan and Kaiser (2005), which identified traits indicating prosociality (e.g., modesty, humility, and integrity) and group-beneficial skills (e.g., decisiveness, competence, and vision) as the most important characteristics of successful leaders. With regard to these prosocial traits, note that Hogan and Kaiser (2005) define integrity as “keeping one’s word, fulfilling one’s promises, not playing favorites, and not taking advantage of one’s situation” (p. 173). Integrity is thus essentially synonymous with trustworthiness, which is an essential trait in a reliable reciprocal partner (Price, 2006a; Rilling and Sanfey, 2011). The emphasis on modesty and humility suggests that followers prefer leaders who are not overly self-centered, which would allow them to focus more on the interests of followers (Price and Van Vugt, in press). Indeed, other results from the GLOBE survey indicate universal distaste for traits associated with leadership that is self-serving and unconcerned with the interests of followers (e.g., dominance, selfishness).

This aversion to overly self-centered leadership is the flip side of the preference for prosocial leaders: leaders are reviled if they control groups in a manner that benefits themselves while harming followers (Tooby et al., 2006). A recent event that epitomizes this principle is the extent to which New Jersey Governor Chris Christie has been excoriated for his perceived role in the “Bridgegate” scandal. Christie’s political allies allegedly orchestrated the partial closure of the Fort Lee bridge from New Jersey to New York – the busiest motor-vehicle bridge in the world – in order to punish Fort Lee’s mayor for not endorsing Christie’s candidacy (Kleinfeld et al., 2014). The lane closures caused traffic chaos and imposed large costs on Christie’s own electorate, all for the intended purpose of generating a narrowly selfish and relatively trivial benefit for Christie. It is also worth considering, in this context, that the extreme levels of income inequality that can obtain in modern businesses may be perceived by followers as exploitative failures of reciprocity. CEOs in the United States between 2000 and 2013, for example, made 200–400 times as much as the average worker (Mishel and Sabadish, 2013), a level of inequality that far exceeds those observed in hunter-gatherer societies (Smith et al., 2010). Leaders who accept salaries that are massively higher than those of followers may be seen as hoarding group resources for their own selfish ends (Price and Van Vugt, in press; Van Vugt et al., 2008a).

Finally, note that although people universally prefer “fair” leaders (Den Hartog et al., 1999), this universality probably masks some important underlying variance. Because different types of followers prefer different kinds of fairness, it will often be difficult for a leader to achieve reciprocity with all followers simultaneously. Whereas some followers may perceive social equality as maximally fair, others may see some forms of inequality as maximally fair, for example, inequality that results from socially sanctioned competition (such as better-qualified job candidates competing successfully for higher-paying jobs). Increased approval of inequality and competition is expressed by individuals who are better-positioned to win competitions for status and resources, such as the highly educated, the wealthy, and members of ethnic majorities (Ritzman and Tomaskovic-Devey, 1992; Pratto et al., 2006; Kunovich and Slomczynski, 2007).

Other traits associated with approval of inequality and competition may be more comprehensible in terms of ancestral environments than modern ones. For example, men who are relatively muscular are more likely to approve of social and economic inequality (Price et al., 2011), particularly if they are relatively wealthy (Petersen et al., 2013; however, this research also found that relatively muscular men are *less* approving of inequality if they are relatively poor). Muscular men are also more likely to endorse aggressive methods of conflict resolution, including war (Sell et al., 2009; Price et al., 2012). These preferences may have been adaptive ancestrally, when muscularity was relatively key to success in social competitions, but seem less rational in modern contexts in which such success has more to do with education and technology. Thus, the kind of “fair” leaders that followers prefer may sometimes be mismatched with aspects of modern environments.

### PREDICTION 5: FOLLOWERS WILL PREFER LEADERS WHO DELIVER INGROUP ADVANTAGE

Ancestrally, one of the most vital benefits leaders could provide was expertise in matters of intergroup competition, and in modern environments, followers prefer leaders who are perceived as ingroup members and strong representatives of ingroup interests (Hogg, 2001). At times when the ingroup is threatened by an external enemy, members will most exhibit this pro-ingroup bias and be most supportive of their leader (the “rally effect”; Van Vugt et al., 2008a). The rally effect benefits followers, who gain security via their increased willingness to cooperate with the leader’s efforts to organize them against the enemy, and it also benefits leaders, who gain status due to their increased ability to benefit the group (Van Vugt and De Cremer, 1999). Because the rally effect enhances the leader’s status, there is the potential for abuse; leaders could exaggerate or provoke an external threat in order to consolidate their own power (Price and Van Vugt, in press).

On the other hand, leaders can also use the rally effect in a less self-serving and more group-beneficial way. If followers are persuaded by a leader of a great need to beat a competitor, they may cooperate particularly effectively: experimental evidence suggests that groups are more cooperative and productive when they perceive a competitive threat from an external group (Van Vugt et al., 2007; McDonald et al., 2012). Note, however, that this tendency appears to be stronger in males than females, which suggests that it evolved in conditions of male coalitional violence (Van Vugt et al., 2007). This suggestion is also made by evidence that people who are experiencing or have experienced life during wartime tend to play economic games in a more cooperative and egalitarian manner (Gneezy and Fessler, 2012; Bauer et al., 2014).

### PREDICTION 6: DOMINANT LEADERSHIP EMERGES WHEN FOLLOWERS LACK EXIT OPTIONS

Service-for-prestige predicts that leaders benefit by adopting a more dominant and coercive leadership style when they can get away with it, because this saves them the costs of delivering benefits to followers. Leaders should be able to get away with this more in modern contexts in which their bargaining power relative to followers is increased for whatever reason, for example, due to it becoming institutionally normalized (Henrich and Gil-White, 2001) in a way that increases follower dependence (Emerson, 1962). One of the most important ways in which leaders’ relative power will be increased in modern societies, however, is if followers have reduced power to exit groups. It was noted above that leadership in small-scale societies seems to become more dominant and less reciprocal when followers have better exit options, and it has long been suggested that a similar pattern exists in industrialized societies, with leadership becoming more autocratic when members have fewer exit options (cf. Hirschman, 1970).

In consideration of the above, service-for-prestige expects that leaders of modern organizations will more likely adopt a dominance-based leadership style when employees are less able or willing to leave their jobs, or to otherwise reject or retaliate against non-beneficial leaders (Price and Van Vugt, in press). In such situations, it will be less necessary for leaders to pay the costs of providing benefits to followers in order to compel them to remain in the organization. This may explain why employees with better exit options tend to receive a greater share of organizational rewards (Rusbult et al., 1988). If leaders adopt a coercive leadership style when their followers do possess good exit options, they will likely lose followers: in experimental research by Van Vugt et al. (2004), members defected more from groups if they were led by autocratic-style instead of democratic-style leaders.

When followers lack exit options, and thus have reduced bargaining power to demand reciprocity with leaders, a more comfortable niche is created for leaders who are concerned only with maximizing their own power and who have no genuine regard for follower interests (Price and Van Vugt, in press). Such toxic leadership may be exhibited by those with high scores on at least one of the “dark triad” traits of Machiavellianism, narcissism, and psychopathy (Paulhus and Williams, 2002; Van Vugt and Ahuja, 2010). Such traits appear to be more prevalent among corporate leaders than among the general population (Babiak et al., 2010), and employees who serve under leaders high in such traits report reduced well-being and job satisfaction (Mathieu et al., 2014).

### PREDICTIONS 7 AND 8: DISRESPECTFUL FOLLOWERS OF BENEFICIAL LEADERS WILL ATTRACT SOCIAL PENALTIES (PUNISHMENT AND/OR SOCIAL EXCLUSION) FROM OTHER FOLLOWERS, WHEREAS DISRESPECTFUL FOLLOWERS OF NON-BENEFICIAL LEADERS WILL ATTRACT SOCIAL REWARDS (ENHANCED REPUTATION AND PRESTIGE) FROM OTHER FOLLOWERS

We conclude this section by looking at some of the novel predictions that service-for-prestige is able to make because it regards leader–follower reciprocity as a collective action problem. As noted above, service-for-prestige predicts that leaders provide public goods to followers in exchange for prestige, which followers must collectively supply. As in any collective action, this presents a free rider problem: a follower who took leader-provided benefits without paying the costs of respect (by going out of one’s way to promote the welfare and interests of the leader, including by deferring to and cooperating with the leader) would be advantaged over respectful followers. In order to solve this problem, this advantage would have to be neutralized or reversed. As in other collective actions, this neutralization/reversal would be expected to occur via the imposition of social penalties on the free rider, in the form of punishment (e.g., physically harming or appropriating resources from the disrespectful follower) and/or social exclusion (i.e., preventing the disrespectful follower from participating in advantageous social and cooperative interactions; Fehr and Gächter, 2002; Price and Johnson, 2011). Thus, a general prediction of service-for-prestige is that disrespectful followers of beneficial (i.e., public-good-provisioning) leaders will tend to attract social penalties from other members of their group. Note that because such a penalty would represent a cost imposed on not only a free rider in a collective action but also a cheater in a reciprocal exchange (with the leader), it could take the form of negative indirect reciprocity (Alexander, 1979; Nowak and Sigmund, 2005), whereby one follower imposes costs on another for failing to engage in reciprocity with the leader.

In considering the various ways by which social penalties could be imposed on disrespectful followers, it is important to keep in mind that to the extent that a penalty is costly to impose, it could lead to a second-order free rider problem (Yamagishi, 1986; Boyd and Richerson, 1992). Second-order free riders in this situation would be followers who paid the costs of respect, but who did not penalize disrespectful followers; they would obtain the benefit produced by these penalties (i.e., the leader’s continued motivation to produce public goods), but by avoiding the costs of administering these penalties, they would acquire higher net benefits than followers who paid to allocate both prestige and penalties. There are a variety of ways in which evolution could overcome the recursive nature of free rider problems and there is no consensus about how it does so (Price, 2003). However, researchers do agree that evolution overcomes these problems somehow (Boyd et al., 2003; Barclay, 2006; Bowles and Gintis, 2011).

Researchers also agree that the penalties used to solve these problems can take the form of direct punishment, and that cooperators in collective actions tend to experience punitive sentiment toward free riders (Price et al., 2002; Mathew and Boyd, 2014). Punitive sentiment leads those who experience it to support and advocate the punishment of free riders, and may lead them to administer this punishment themselves (Fehr and Gächter, 2002; Price, 2005). It is therefore plausible that respectful followers experience punitive sentiment toward disrespectful followers, and act on this sentiment either by punishing these followers themselves, or else by advocating and supporting the punishment of these followers by other group members. This punishment could be administered by one specific member (O’Gorman et al., 2009), including the leader him/herself. Even in situations in which leaders impose such penalties themselves, we would expect for followers to experience punitive sentiment, as it would lead them to lend political support to the leader’s punitive actions. Punishment could also be administered in a coordinated manner by more than one member; coordinated punishment could reduce the per capita costs of punishment and thus mitigate the second-order free rider problem (Boyd et al., 2010; Guala, 2012).

The social penalties of free riding do not have to involve direct or explicit punishment, and in both small-scale and industrialized societies they may take the form of informal social sanctions that lead to reputational damage (Fried, 1967; Falk et al., 2005). Among hunter-horticultural Shuar, for example, villagers who are perceived as being less respectful of a popular leader are themselves respected less (Price, 2003). The main costs of such reputational damage may involve exclusion from advantageous social interactions (Barclay and Willer, 2007; Sylwester and Roberts, 2010; Baumard et al., 2013). In modern organizations, employees who act disrespectfully toward popular leaders may be sanctioned by other employees via social exclusion processes that are facilitated by gossip (Barkow, 1992; Williams, 2007).

Note that the prediction of social penalties (punishment and/or social exclusion) for disrespectful followers only applies to situations in which leaders are providing followers with public goods, because this is the only context in which followers will need to engage in reciprocity with leaders by collectively providing them with prestige. If followers do not perceive that a leader is providing them with benefits, they should not be motivated to generate prestige for that leader, nor to impose costs on other followers who fail to generate prestige. On the contrary, if the leader is unpopular, followers should tend to regard disrespectful followers in a positive light. Leaders will be unpopular if they are seen as failing to provide public goods for one reason or another, for example, because they are incompetent or exploiting followers for their own narrowly selfish ends (Tooby et al., 2006), and followers face the problem of how to collectively strip such leaders of status. A disrespectful follower of an unpopular leader risks retaliation from the leader, and so will be seen by co-members as a selfless and prestige-worthy contributor to the public good: if you brave the wrath of Darth Vader, you will become a hero to the rebellion. Thus according to service-for-prestige, rescinding status from a non-beneficial or exploitative leader, just like supplying it to a beneficial one, is a collective action problem (Price and Van Vugt, in press). Accordingly, service-for-prestige predicts that disrespectful followers of such leaders will attract social rewards from other followers. These rewards will come in the form of enhanced prestige, which like all forms of prestige (as discussed above) should afford increased access to material, reproductive, and/or social resources.

## DISTINGUISHING SERVICE-FOR-PRESTIGE FROM OTHER LEADERSHIP THEORIES

### NOVEL PREDICTIONS OF SERVICE-FOR-PRESTIGE

A Lakatosian view (Lakatos, 1978) suggests that a progressive scientific theory will make not only correct predictions shared by other theories, but also novel correct predictions. Some predictions of service-for-prestige presented above may be shared with many leadership theories (e.g., that followers will prefer leaders who are intelligent, competent, and able to deliver ingroup advantage). We included these predictions not to suggest that they are unique to service-for-prestige, but in order to demonstrate that service-for-prestige is consistent with a broad range of well-supported observations about leader–follower relations. These are observations that any useful theory of leader–follower relations should explain, even if other theories can explain them as well. In order to judge the added value of service-for-prestige, however, we must focus on whether it makes any novel predictions.

Because of its evolutionary foundations, service-for-prestige makes some predictions that are not shared by most non-evolutionary theories. These include predictions related to mismatch theory, such as that followers will judge leaders based on characteristics that are more relevant to small-scale societies than to modern organizations (e.g., physical formidability). However, most of these mismatch-related predictions are also made by the evolutionary leadership theory presented by Van Vugt and Ahuja (2010). What truly distinguishes service-for-prestige is not its evolutionary foundations *per se*, but rather its assumption that leader–follower relations evolved via a reciprocal interaction that entailed collective action problems for followers. Thus, the most important novel predictions made by service-for-prestige are those which emanate directly from this assumption, specifically, predictions seven and eight above: disrespectful followers of beneficial leaders will attract social penalties from other followers, whereas disrespectful followers of non-beneficial leaders will attract social rewards from other followers. As far as we are aware, no other theory of leader–follower relations makes not only predictions 1 through 6 above, but also predictions 7 and 8.

### COMPARING SERVICE-FOR-PRESTIGE TO OTHER EVOLUTIONARY THEORIES FOR WHY IT PAYS TO LEAD

Predictions 7 and 8 distinguish service-for-prestige not just from the evolutionary leadership theory mentioned above (Van Vugt and Ahuja, 2010), but also from two other notable evolutionary theories of leadership. Both of these theories address the issue of why, given the costs that leaders must incur in order to generate public goods for followers, it would be adaptive for leaders to lead.

The first of these theories is costly signaling theory, which proposes that acts of providing public goods to one’s group can serve as a valuable opportunity to advertise one’s desirable qualities to potential allies, cooperative partners, and mates (Gintis et al., 2001; Hawkes and Bliege Bird, 2002). For example, the opportunity to lead a hunt might afford one an opportunity to show off attractive traits such as hunting skill, health, and formidability. This advertising could lead to fitness-enhancing social and mating opportunities that would compensate the leader for leadership costs. Although costly signaling offers a plausible explanation for some aspects of leadership, it is distinguishable from service-for-prestige because it does not predict that followers face the collective action problem of generating prestige. Instead, it presumes that the increased social affiliation that a follower offers a leader represents a private good for both leader and follower. This focus on private goods is why costly signaling theory does not make predictions 7 or 8 above (Price, 2003).

The second evolutionary theory of leader compensation could be called the “asymmetric interest” model. From this perspective, leaders of collective actions are compensated by virtue of the fact that they have a greater interest in the success of the collective action than do most followers. Their interest may be greater, for example, because compared to followers they stand to acquire a larger share of the resource produced, or because this resource is inherently more valuable to them (Tooby and Cosmides, 1988; Ruttan and Borgerhoff Mulder, 1999; Hooper et al., 2010). Before examining how this theory is distinguishable from service-for-prestige, it is important to note that some versions of it could in fact be identical to service-for-prestige in everything but name. This would be the case if the leader’s increased interest in the collective action were the result of collectively allocated prestige from followers, for example, if this prestige led followers to jointly and voluntarily grant the leader a relatively large share of the spoils. If the leader’s increased interest in the collective action were not the result of such prestige, however, then this theory would make different predictions than service-for-prestige. Because it would predict that the leader was leading the collective action out of a private interest in collective success, it would not predict that the leader would require any additional compensation from followers (e.g., prestige), nor that followers would face a collective action problem in generating this prestige. Therefore this theory would not even make the prediction that leaders will acquire more prestige than followers, let alone predictions 7 and 8 above.

### COMPARING SERVICE-FOR-PRESTIGE TO EXISTING NON-EVOLUTIONARY LEADERSHIP THEORIES

Service-for-prestige has some important aspects in common with previous exchange theories of leadership that are not explicitly grounded in evolutionary theory (Price and Van Vugt, in press). One of these is leader–member exchange theory (LMX; Graen and Uhl-Bien, 1995), which suggests that leadership quality depends heavily on the quality of social relationships between a leader and individual followers. Transactional theories of leadership are also exchange theories; the exchange process that is usually emphasized in these theories is one in which leaders use punishments and rewards to motivate followers to achieve group goals (Bass, 1991). Interestingly, however, Hollander (1992) suggests that this transaction may take the form of leaders providing benefits to followers in exchange for esteem. Also relevant is servant leadership theory (Greenleaf, 2002; Gillet et al., 2011), which emphasizes that the influence of good leaders stems from their compassion, altruism, moral authority, and ability to benefit followers.

Despite some clear points of similarity with these theories, service-for-prestige possesses several unique attributes (Price and Van Vugt, in press). Unlike servant leadership, service-for-prestige sees the “altruism” of good leaders as something that not only benefits followers, but also ultimately profits leaders (because leaders exchange this altruism for prestige). Unlike LMX, service-for-prestige focuses on relationship quality not in general but specifically in terms on how evolution designed both leaders and followers to achieve an efficient exchange of fitness-relevant benefits. Further, service-for-prestige examines relationships between leaders and groups of followers, whereas LMX focuses on dyadic leader–follower relationships. Only service-for-prestige, therefore, predicts that followers face a collective action problem in generating leader prestige. And unlike either servant leadership or LMX, service-for-prestige attempts to explain not only how leadership can most benefit followers, but also how it can most harm them.

Finally, service-for-prestige can potentially explain not only the transactional, material rewards and punishments provided by leaders to followers, but also the “symbolic” benefits of leadership such as enhanced cohesion and identity; such relatively abstract benefits may plausibly have contributed to the fitness of individual group members in the past, if they improved the group’s solidarity and hence ability to compete for resources with other groups. In other words, symbolic benefits may represent proximate mechanisms that over evolutionary time have enabled leaders and followers to achieve more ultimate (fitness-related) goals. In this respect, service-for-prestige has as much in common with transformational leadership models of leadership (Bass, 1991, 1998) as with transactional or social identity models of leadership (Price and Van Vugt, in press).

## SERVICE-FOR-PRESTIGE AND SOCIAL NEUROSCIENCE

Service-for-prestige proposes that voluntary leader–follower relations involve many of the same behaviors that are involved in cooperative interactions more generally, such as reciprocal exchange, collective action, and punishment of non-cooperators. A growing body of social neuroscience research focuses on these behaviors, especially exchange and punishment (Rilling and Sanfey, 2011). Most of this research has examined cooperation in dyadic contexts and relatively little has focused on group behaviors, which to some extent limits the direct applicability of this research to the group contexts of leadership. However, the science is developing rapidly, and interest is now turning to the neuroscience of group cooperation as well (Zak and Barraza, 2013).

As social neuroscience is uniquely poised to reveal the specific neural systems that are involved in cooperative behaviors, it may ultimately provide the best methods for testing some of the most fundamental claims of service-for-prestige. One such claim is that voluntary leader–follower exchange evolved as an elaborated form of dyadic cooperation, which allowed for reciprocity to occur between an individual and multiple group members. If correct, then the same core neural systems involved in dyadic cooperation are likely also involved in voluntary leader–follower exchange. If social neuroscience could shed light on the validity of this assumption, it would contribute to the resolution of a debate among evolutionary researchers, some of whom claim that cooperation between an individual and multiple group members is likely to involve direct reciprocity (Price, 2003, 2006a; Tooby et al., 2006) and some of whom claim such cooperation could evolve only via completely different processes such as cultural group selection (Boyd and Richerson, 1988; Henrich, 2004; Bowles and Gintis, 2011).

If service-for-prestige is correct to expect that neural systems that evolved to enable dyadic reciprocity are also fundamental to leader–follower exchange, then there already exists a fairly large body of social neuroscience research that is relevant to service-for-prestige. There has been quite a bit of neuroscience-related research on behavior in the ultimatum game, for example, and this game can be interpreted as a simple form of leader–follower exchange. The ultimatum game is a two-player economics experiment involving a proposer and a responder. The proposer is given a sum of funds and decides how much to offer to the responder. If the responder accepts the offer, then the deal goes through. For example, if the proposer offers 40% of $10 to the responder and the responder accepts, then the proposer keeps $6 and the responder takes home $4. If the responder rejects the offer, however, then both players get nothing. The “rational” decision for responders is to accept any offer greater than 0, even if it is very low, because if they reject they will always get 0. By the same token, the rational decision for proposers would be to offer the lowest possible amount above 0. However, research shows that responders tend to reject offers that are too far below 50%, and that proposers’ offers tend to be close to 50% (Bowles and Gintis, 2011). The proposer can be seen as the leader, and the proposer characteristics that cause cooperation to fail in the game – that is, selfishness and unfairness – are those that followers associate, as noted above, with dominant and “bad” leadership (Den Hartog et al., 1999). A rejected offer in the ultimatum game, therefore, is essentially similar to a case of failed leadership.

Most studies on the neural and hormonal correlates of behavior in the ultimatum game are concerned with responder behavior, but a few focus on proposers. For instance, one study shows that patients with damage to the ventromedial prefrontal cortex (VMPFC), a condition associated with impaired concern for other people, made more selfish proposals. Compared with a control group these patients offered significantly less and seemed less motivated by the emotion of guilt (Krajbich et al., 2009). These results suggest that such individuals would be less likely to emerge as successful leaders in the context of service-for-prestige exchanges. Other research found that men who received a small dose of testosterone, a hormone associated with aggression and status competition, made more unfair offers compared to men who received a placebo (Zak et al., 2009; cf. Burnham, 2007, who found no significant relationship between circulating testosterone levels and ultimatum game offers in men). Considering that unfair offers are more likely to be rejected, this result suggests that some neurological effects of testosterone in men may reduce effectiveness as a service-for-prestige leader (however, other effects of testosterone, such as those increasing physical formidability, may increase perceived suitability for leadership). A recent study among a sample of managers shows that baseline testosterone levels correlate positively with a more dominant leadership style, and negatively with a laissez-faire leadership style (Van der Meij et al., unpublished data).

Other studies have been conducted to examine the neural correlates of responder behaviors in the ultimatum game, which can be conceived of as a measure of followership. Data on patients with brain damage, especially in the VMPFC, show that they are more likely than healthy participants to reject offers that are deemed unfair (Koenigs and Tranel, 2007). This brain area is thought to be important for emotion regulation. Other studies have suggested that people with higher levels of serotonin – which has been linked to aggression, hostility and impulsivity – are less likely to accept unfair offers from proposers (Crockett et al., 2008; Emanuele et al., 2008). Higher levels of circulating testosterone have also been linked to increased rejection of unfair offers (Burnham, 2007). Taken together, these studies on VMPFC damage, serotonin and testosterone support the view that rejection of unfairness is related to impulsivity and negative emotion, and that individuals who are less able to regulate negative emotion will be less likely to emerge as followers in unequal leader–follower relationships. On the other hand, better ability to regulate negative emotion might make one more likely to accept unequal (e.g., highly dominant) leadership. An fMRI study by Kirk et al. (2011) compared ultimatum game behavior in people who regularly perform Buddhist meditation (entailing training in the regulation of negative emotion) and a control group. Meditators accepted unfair offers more often than controls, and displayed reduced activity in the anterior insula, an area associated with the emotion of disgust.

Studies have also begun to indicate the general brain regions involved with the punishment of non-cooperators in dyadic trust game contexts (de Quervain et al., 2004; Singer et al., 2006). With increased methodological precision, and as studies broaden their focus to include punishment of free riders in collective actions, we will become increasingly able to answer punishment-related questions raised by service-for-prestige, such as: when a follower who respects a leader perceives another follower to be disrespecting that leader, does he or she experience punitive sentiment that is similar (in terms of the neural systems involved) to the anti-free-rider punitive sentiment experienced by high-contributing members of other kinds of collective actions (Fehr and Gächter, 2002; Price et al., 2002)? Such results will shed additional light on debates between evolutionary researchers, mentioned above, about the extent to which leader–follower relations were designed by the same evolutionary processes that enable reciprocity and collective action in other cooperative contexts, as opposed to being designed by qualitatively different processes such as cultural group selection.

## CONCLUSION

Service-for-prestige does not claim that either kind of leader–follower relationship described above – prestige-based reciprocity or dominance-based coercion – is more “natural” than the other; people are adapted for both kinds of interaction. However, reciprocity clearly involves the greater degree of mutual benefit. Unlike coercion, reciprocity allows followers to act on their leader preferences, and collectively award prestige to people who, via their ability to benefit followers, they deem worthy of leadership roles. Reciprocity is also more closely associated with what most would consider “good” leadership (Den Hartog et al., 1999), that is, leadership that most helps followers to achieve their shared goals, as opposed to primarily serving the narrow interests of the leader (Price and Van Vugt, in press).

There is also much that service-for-prestige does not explain about the evolution of leadership. Most importantly, as noted above, it does not explain why leadership has evolved in so many non-human species, nor why it first appeared in the human lineage. Leadership has evolved in many species to solve problems related to information-sharing and coordination problems (King et al., 2009), and these were probably its initial functions among human ancestors as well (Van Vugt and Kurzban, 2007; Van Vugt et al., 2008a). However, we suggest that humans, much more than any other species, have been able to use reciprocity to enhance the benefits of leadership, by providing leaders with prestige in exchange for services that would otherwise have been too costly for leaders to provide. Our theory applies to humans much more than any other species, because humans are uniquely well-adapted for sophisticated cooperative behaviors such as collective action and various forms of reciprocity (Tooby and Cosmides, 1996; Hammerstein, 2002; Tooby et al., 2006; Bowles and Gintis, 2011).

By considering the kinds of problems that evolution had to solve in order to enable leaders and followers to interact adaptively in human ancestral environments, service-for-prestige is able to make novel predictions about leader and follower behaviors in modern environments. Service-for-prestige also promotes a synthesis of the theoretical frameworks of positive reciprocity and collective action. Although each of these frameworks has had an enormous independent influence in behavioral science, they are thought of by many evolutionary researchers as distinct and non-overlapping (Boyd and Richerson, 1988; Henrich, 2004; Bowles and Gintis, 2011). We suggest, as others have previously (Price, 2003, 2006a; Tooby et al., 2006), that reciprocity and collective action are not as distinct as these researchers suggest, and that syntheses of these theories will enable more predictive evolutionary theories of complex human cooperation. Social neuroscience is in a unique position to help resolve debates about the extent to which these theories can in fact be integrated. If service-for-prestige is correct, then many of the same neural systems involved in cooperative behaviors such as reciprocity, collective action, and free rider punishment should be similarly involved when these processes occur in the specific context of leader–follower relations. Neuroscientific methods are already proving useful in identifying and illuminating the nature of these systems, and as they become increasingly precise, they will become increasingly essential for answering fundamental questions about how humans are adapted for leadership, followership, and indeed all forms of social behavior. More frequent occasions for interaction between evolutionary social psychologists and social neuroscientists, such as that provided by this special issue, will enable us to take full advantage of these new research opportunities.

## Conflict of Interest Statement

The authors declare that the research was conducted in the absence of any commercial or financial relationships that could be construed as a potential conflict of interest.
